# A qualitative study on the impact of the 2016 US election on the health of immigrant families in Southeast Michigan

**DOI:** 10.1186/s12889-019-7290-3

**Published:** 2019-07-15

**Authors:** Paul J. Fleming, William D. Lopez, Hannah Mesa, Raymond Rion, Ellen Rabinowitz, Richard Bryce, Monika Doshi

**Affiliations:** 1 Department of Health Behavior and Health Education, University of Michigan School of Public Health, 1415 Washington Heights, Ann Arbor, MI 48109-2029 USA; 2 Packard Health, Ann Arbor, MI USA; 3 Washtenaw Health Plan, Ypsilanti, MI USA; 4 Community Health and Social Services (CHASS) Center, Detroit, MI USA

**Keywords:** United States, Michigan, WIC, Trump, Deportation, FQHC

## Abstract

**Background:**

Given the anti-immigrant rhetoric and policy proposals by President Donald Trump during the 2016 presidential campaign and afterwards, his election to president in November 2016 and subsequent policy changes has affected immigrant families. In this study, we aim to better understand how post-election policy change may have impacted the health and well-being, including health and social service utilization, of Latino immigrants in Southeastern Michigan.

**Methods:**

We conducted 28 in-depth interviews with frontline staff at two Federally Qualified Health Centers and a non-profit agency. These staff had intimate knowledge of and insights into the lived experiences of the mixed-status immigrant families they serve. The interviews were audio recorded, transcribed, and analyzed thematically.

**Results:**

Our findings show three major themes: (1) An increased and pervasive fear of deportation and family separation among mixed-status immigrant clients, (2) The fear of deportation and family separation has resulted in fractures in community cohesion, and (3) Fear of deportation and family separation has had an impact on the healthcare utilization and health-related behaviors of mixed-status families. Staff members report that these three factors have had an impact on physical and mental health of these immigrant clients.

**Conclusions:**

These results add to previous literature on the effect of immigration policies on the health and provide key insights for interventions to improve the health of immigrants within this socio-political environment.

## Background

The 2016 national election in the United States catapulted immigrants and immigration policies to the forefront of the collective public consciousness as politicians debated whether or not to include or exclude immigrants—particularly immigrants from non-European countries—in U.S. society. The immigration-related rhetoric and policy changes following the 2016 US. presidential election have been frequent and have raised the question of how these changes have impacted the health of immigrants in the US. This paper aims to explore this question by reporting on in-depth interviews conducted with health and social services staff who work closely with immigrant families in Southeastern Michigan.

### Immigration-related rhetoric, policies, enforcement, & health

While enforcement of immigration laws has historically been part of federal policy, the federal government has recently broadened the criteria for immigrants it prioritizes for arrest and deportation. While immigrant detention and deportation under the Obama administration focused mostly on ‘serious criminals’, the standard practice under the Trump administration is to target any undocumented immigrant who has broken the law (including entering the U.S. unlawfully) and to detain any other immigrants who are nearby at the time of arrest [1]. Since President Trump took office, his administration has expanded the number of Immigration and Customs Enforcement (ICE) officers, and there was a 40% increase in detention of undocumented immigrants in the first year of the Trump presidency [1].

Medical providers are concerned that these expanded immigration enforcement efforts—as well as rhetoric and other policies—have created an anti-immigrant climate that will result in a ‘chilling effect’ on undocumented immigrant’s willingness to receive health and social services [2]. This is particularly problematic given that immigration policy enforcement actions have been shown to contribute to poor health among Latino immigrants [3–8]. Page & Polk speculate that the anti-immigrant climate may cause undocumented immigrants to avoid traveling to clinics or giving their information to clinics and government programs because they fear it will increase their likelihood of being detained and deported [2]. Two draft executive orders by President Trump contribute to this concern; one threatens to revoke current ICE policies that protect immigrants from being detained or deported at health care facilities [9, 10] and one would penalize immigrants who have received Medicaid, WIC, or other public benefits in their applications for residency and citizenship [10]. Though they are still under consideration, the threat contributes to the anti-immigrant climate. Taken together, these policies and threatened actions have created a hostile environment that potentially may cause immigrant families to avoid health-care settings or enrolling in government programs.

Prior research has shown that undocumented immigrants have less access to health care than documented immigrants and non-immigrants, primarily due to the combination of health policies, immigration policies and enforcement, and discrimination [11]. Undocumented immigrants are not provided access to health insurance exchanges or Medicaid/Medicare through the Affordable Care Act. Thus, many depend on public clinics or emergency rooms for health care. One study examined the impact of Alabama’s 2011 Alabama Taxpayer and Citizen Protection (House Bill 56)—a bill that requires proof of lawful U.S. residence to receive state and local public benefits except those protected by federal law—on use of county public health clinics in Jefferson County, Alabama. They showed that visits to county public health clinics decreased by nearly 25% among Latino adults after the law went into effect, largely due to the fact that undocumented Latinos did not have proof of lawful U.S. residence [12]. Discrimination also plays a role: among undocumented immigrants, individuals who reported experiencing general discrimination were less likely to have access to health care providers than those who did not experience discrimination [13]. Together, these findings suggest that the recent increase in immigration enforcement efforts could have a negative impact on health-seeking behaviors by immigrants.

Given that undocumented immigrants do not have access to the Affordable Care Act health insurance exchanges or Medicaid/Medicare [14], Federally Qualified Health Centers (FQHCs) are one of the only affordable options in the formal health care system for undocumented immigrants. As part of FQHC’s eligibility to receive federal funding, they are required to help clients obtain Medicare/Medicaid and to offer primary care services at a sliding-scale fee to uninsured and underinsured patients. Additionally, local non-profits or health departments often provide low-cost or free health services that immigrants are able to use. Research conducted in 2013–prior to the most recent changes in immigration enforcement and the upsurge in anti-immigrant rhetoric–found that FQHCs were a key resource for undocumented Latino immigrants in the western U.S. [15]. Additionally, this research found that because of the FQHC’s long-standing outreach efforts, undocumented immigrants that feared deportation still attended health services. Hoerster et al. [16] similarly showed that FQHCs play a vital role in the health of immigrants in the southwestern U.S., with some incorporating outreach programs to help curtail concerns related to deportation while increasing access to care among their immigrant clients.

Rapid policy shifts and anti-immigrant rhetoric by President Donald Trump during the 2016 presidential campaign and afterwards may have had an impact on the health and well-being of immigrant families. In this study, we aim to understand how the 2016 presidential election impacted the health and social welfare among immigrant communities in Southeastern Michigan in the year after the election. To understand this issue, we chose to interview health and social service workers because they have a view of both the impacts on a broad range of immigrant clients and impacts on the health and social care institutions where they work.

## Methods

We use data collected as part of an ongoing research collaboration informed by the principles of community-based participatory research (CBPR) [17]. The project was initiated in response to questions initially raised by our partners, a Federally Qualified Health Center (FQHC) in Detroit, MI, an FQHC in Washtenaw County, MI, and a non-profit agency housed at the Washtenaw County Health Department that provides healthcare coverage and other social services for immigrant clients. These three organizations all serve immigrant clients and have anecdotally noticed changes since the 2016 election and wanted to understand how they might need to modify their existing services to better meet the needs of their immigrant clients. The research presented in this paper is the result of the first phase of this research collaboration between these organizations and the University of Michigan. All study procedures were approved by the University of Michigan Institutional Review Board.

### Study locations

Approximately 130,000 undocumented immigrants live in Michigan, with most residing in the urban/suburban areas of southeastern Michigan [18]. Many of these undocumented immigrants live in ‘mixed-status’ families, with either a partner or child(ren) who are U.S. citizens or legally authorized to reside in the U.S. While many do not think of Detroit as a ‘border town,’ the Detroit metro area is home to one of the busiest land border crossings between the U.S. and Canada. As a result of the juxtaposition between a large metro and an expansive international border, both Customs and Border Patrol (CBP) and Immigration & Customs Enforcement (ICE) are active in the region.

We conducted our research at three locations in Southeast Michigan: a Detroit FQHC, Washtenaw County FQHC, and a Washtenaw County non-profit. Detroit is the largest city in Michigan and home to a long-standing population of Mexican immigrants as well as a newer influx of Central Americans. The Detroit FQHC is located in a predominantly Latino neighborhood and provides clinical and social serves to residents of the neighborhood. Given their large Spanish-speaking client population, nearly all staff are bilingual and many are Latino. Washtenaw County is about 45 miles west of Detroit and home to the town of Ann Arbor, Ypsilanti, and others. About 11% of Washtenaw County residents are foreign-born and while most immigrants are from Asia, most undocumented immigrants are from Latin America. The Washtenaw County FQHC provides clinical and social services to a wide range of populations with immigrants comprising about a quarter of their client population. Staff at both FQHCs comprise of clinical, administrative, and social service workers personnel. The non-profit partner helps people access health insurance, including a safety net program that reaches most of the county’s undocumented residents. They are housed in the Washtenaw County Health Department and while they serve all members of the county, the vast majority of their clients are immigrants. Staff at the non-profit have extensive experience helping immigrant families access health coverage and access county health and social services.

### Study participants, data collection, and analysis

Our data collection and analytic process were informed by the interpretive description approach [19] that is focused on pragmatic knowledge creation from qualitative data to improve health outcomes [20]. In our case, we aimed to produce knowledge related to the impacts of the 2016 election in order to improve health and social services available to immigrants. Practically, this meant that we sampled people who could provide the most useful information to the research question, developed an interview guide in collaboration with leadership at these institutions to help provide useful information, and analyzed the data using strategies that kept the focus on questions of ‘what is happening here?’ and ‘what am I learning about this?’ [19, 20].

In this paper, we focus on 28 in-depth interviews with staff members at these three agencies because they provide a comprehensive view of the impacts across their institution, across their community, and across different immigrant families. For the staff interviews, we utilized in-depth interviews because we sought to understand how each staff member experienced the post-election period in terms of the clients they interacted with and the specific services they were delivering. With the help of leadership at each organization, we recruited staff members for in-depth interviews. We used a convenience sample to talk with staff members who had significant experience working with immigrant clients. At each location, the research team brainstormed the different roles within the organization that interacted with immigrant clients and the lead person at each organization referred staff members in each of those roles to to the university-based members of the research team for interviews. Once referred, they went to a private space, were explained more information about the project, assured that participation or non-participation would not affect their employment. Interviewers made it clear that the information they shared—including if they declined the interview—would not be shared with their employer or colleagues in a way that could identify the person. All participant underwent underwent written informed consent procedures.

All interviews were conducted between April 2018 and August 2018 by the first and second author. At the time of the research, the first author was a faculty member and the second author was postdoctoral fellow in the field of public health. Both are experienced qualitative researchers with a strong grounding in community-based research collaborations focused on the health of immigrants. The first author had little prior experience in this specific region whereas WDL had been working in Washtenaw County and Detroit on immigration research for the prior decade.

The interviewers used an interview guide for the conversations but often adapted questions to the particular experiences of the interviewee. The interview guide was first developed by PJF and WDL based on conversations with leadership at each institutions and then was reviewed, edited, and approved by the leadership at each institution. Example questions in the guide included: “What do you perceive as the major facilitators to immigrants accessing care at your organization?” “How do you perceive the political climate as affecting their ability to access care?” and “What changes have you seen in your work over the past X number of years?”

### Data analysis

Data analysis began as data collection occurred. The interviewers took notes and had discussions about the themes that were emerging during the interviews. These post-interview discussions allowed the interviewers to iteratively adapt the interview guide and probe on emerging and important topics in order to reach saturation of themes.

All interviews were audio-recorded and transcribed verbatim by research assistants. Our team read through transcripts and listened to audio while taking notes of emerging findings. For each interview, the first author wrote a narrative summary related to the research question: how did the 2016 presidential election impact the health and social welfare of immigrant communities in Southeastern Michigan? Based on those narrative summaries and several analysis meetings to discuss the transcripts, we iteratively developed a codebook of 24 codes and applied them to the transcripts using NVivo version12. Our codebook included codes such as 2016 Election, Generalized Fear, Police/ICE/CBP, Transportation, and Facility Environment. After the transcripts were coded, we examined coding outputs to better understand the various perspectives of participants related the research question. When examining the coding output, we wrote memos on emerging key themes that were salient to the research questions.

After finalizing preliminary results from the staff interviews, we wanted to verify that the perspectives shared by the 28 staff members interviewed were confirmed by staff members at these sites who were not interviewed. First, we presented the findings to two separate groups of staff--one in Washtenaw County and one in Detroit--to verify that our findings reflected their experiences and perspectives working with immigrant clients during this period. In the results section, we present these themes using illustrative quotes from the transcripts.

## Results

We interviewed a total of 28 staff members across the three sites: FQHC in Detroit (*n* = 12), FQHC in Washtenaw County (*n* = 5), Washtenaw County non-profit (*n* = 12). Twenty-three were bilingual English-Spanish speakers and 18 self-identified as Latino. The staff roles interviewed included medical providers, medical assistants, receptionists, enrollment specialists, patient advocates, and others.

Our analyses identified three major interrelated themes in the context of our research question: 1. An increased and pervasive fear of deportation and family separation among clients in mixed-status families; 2. fear because of the anti-immigrant climate has resulted in fractures in community cohesion; and 3. fear has had an impact on healthcare utilization and other health-related behaviors among mixed-status families in an effort to prevent deportation and protect their families. In this section, we detail each of these themes and provide illustrative quotes using pseudonyms.

### Increased and pervasive fear

Universally, staff members discussed that their clients who are part of mixed-status families are experiencing more fear and anxiety related to possible deportation than before the election. Isabel, a staff member at the Detroit FQHC, succinctly summed up staff perspectives of this increased fear: “They’re fearful. They are so much more fearful now than they were 2,3,4,5,10 years ago…huge huge impact, huge difference.” Staff members across roles, location, and ethnicities spoke about how the 2016 election and the inauguration of President Trump caused mixed-status families to be fearful that a family member would be suddenly detained and deported. Most participants said that this fear has lasted, but was most acute in the immediate period after the election. Vanessa, a family medicine doctor at the Washtenaw County FQHC said:

*“The time between the election and the actual inauguration, I think that was the worst cause there was a lot of ‘we have no idea what’s going to happen’ and just a lot of fear. People would just come in and say ‘I’m afraid’.”*

Some staff members questioned whether the fear was due to an actual increase in deportations or attributable to anti-immigrant rhetoric and uncertainty related to policy proposals (that is, policies that had yet to be enacted). Brian, a family medicine doctor at the Detroit FQHC reflects some of these ideas in his comments. He initially points out that anti-immigrant sentiment is driving the pervasive fear: “the rhetoric that comes out from Donald Trump, I think the patients definitely feel it…it’s not even just the ones that are undocumented, I think the community feels it.” Importantly, Brian notes that he is seeing this fear across all his patients, not just those who are undocumented. In contrast, Brian said that he knew many patients who were deported during President Obama’s tenure and that certainly caused some fear in the community; however, he perceived that the impact was narrower under President Obama because immigrants who did not have a criminal record did not feel targeted to the same extent. He goes on to share that now “it just doesn’t seem like there’s any rhyme or reason” and that any immigrant could be a target. He continued: “What makes me more nervous is that the rhetoric creates fear but now the inconsistency creates more fear…[it’s] paralyzing.” Many other staff members shared this same sentiment that deportations are not new in the Trump era, but that the anti-immigrant rhetoric from politicians and uncertainty of policy decisions is driving a lot of the increased fear in their clients.

### Fractures in community cohesion

Another impact of the 2016 election is that the pervasive fear caused fractures in the communities where immigrants live. After the election, several staff reported incidents of racist or discriminatory remarks by non-immigrant clients that were aimed to make immigrants feel unwelcome in their community. Gabriella, customer service representative at the Detroit FQHC, noted that the election seemed to increase cases of overt racism or discrimination against Latino clients:

*“A lot more people seem to be coming out more racist and more against the Hispanic communities. So, I think some of them [immigrants] are more like, you know, they don't wanna go out to the stores or do anything where they're gonna have that complication or be like, well, ‘where's your green card?’ or ‘where's this?’ and then they gotta be like, you know, they get nervous. I feel like sometimes, yeah, I think the election affected the community a little bit.”*

Others echoed this idea that immigrants were increasingly being publicly questioned about their belonging in the U.S. Furthermore, several non-profit staff members noted an event where they had to intervene happened at their offices shortly after the election. T, an enrollment specialist at the non-profit said:

*“There was a person there on the phone. They were speaking in their native language… which they had every right to do. They were not loud, they weren’t belligerent, you know, they weren’t causing a scene or being disruptive. But, yet, you have someone else that was from a different background, wasn’t very [pauses], culturally sensitive shall I say.. and she just made snide comments loud about you know people talking in different languages they need to talk, you know, English. And, it’s like, wait a minute, hold up, we accept everyone here no matter what language they speak or what nationality they are or how much money they have. You know that person was in the wrong. It was just wrong.”*

This event reflected that some of the existing racist fractures within this community were augmented and exacerbated by the political climate during the election and afterwards. According to several staff members interviewed, non-immigrants were increasingly willing to verbally harass immigrants who were non-white or spoke another language. Increased threat of such incidents resulted in greater fear among mixed-status families for two reasons: (a) they feared being harassed in public and sought to avoid such instances, and (b) they were concerned that a racist community member could report them to the police or immigration enforcement.

The election and resulting policies also created fractures within immigrant communities that increased fear and anxiety. Cohesion within immigrant communities was affected because people were afraid that associating with someone who has been contacted by ICE would make them more likely to be targeted for deportation. As Jennifer, a patient advocate at the Washtenaw County FQHC describes: *“*So, after a family member has been detained by ICE, that family is seen in a way as tainted. So, their communication and their contact with other community members, family members, friends, they’re are kind of like the plagued family. ‘Oh, ICE has been to that house, we are not going to associate with them anymore.’” Thus, the fear is located within their own community as well. This demonstrates the pervasiveness of this fear as some mixed-status family members perceive that associating with certain neighbors and friends could make it more likely they are deported or separated from their family.

### Impact on healthcare utilization and other health-related behaviors

Because of this exacerbated fear and sense of exclusion, staff reported that immigrant families are adopting certain behaviors to limit the possibility of deportation and family separation. The most frequently discussed change resulting from the fear was that members of mixed-status families were increasingly avoiding social services and clinics. Staff members at each site reported that immediately following the election, there was a decrease in the number of immigrant clients coming for health or social services. Several participants noted that their waiting rooms--previously filled with immigrant clients--were emptier in the weeks after the 2016 election. T, an enrollment specialist at the non-profit, described what she remembered from that time period:

*“Once elections took place and we had a new president in place, you had clients that weren’t coming. I mean, the office was again literally empty, for a couple of weeks at least, because people were afraid to come in. I remember having someone here in tears, I mean literally in tears, because she was afraid to come out, you know, and she finally came. She made that step and she came. But, next she was worried about getting home safe you know*.”

Similar to T, many others noted that the cancellations and no-shows in November 2016–February 2017 were particularly noticeable. They suspected it was related to the uncertainty of what might happen and the immediate policy changes, such as the ban on travel from certain countries, that followed the inauguration. This effect was noticeable across settings, but staff reported that attendance increased and approached average rates over the next few months.

In addition to the immediate post-election effects on behaviors related to seeking health or social services, staff also noted an increase in no-shows or cancelled appointments when an immigration enforcement action had recently occurred in the community. Martha, a receptionist at the Washtenaw County FQHC, recalled a patient who had called her to cancel an appointment:

*“[I] had a lady that she was very honest. She was like, ‘I’m not going out, I heard that, my grandson told me that immigration is doing a round and I’d rather just stay home.’…That kinda like, I never spoke about it, I never recorded anything, that was a shock to me. I was just like, ‘wow’ and she’s like, ‘yeah I’m just going to call back to reschedule.’”*

This exemplifies that clients are primed with fear and an indication that their safety may be threatened—such as immigration enforcement actions nearby--caused them to consider avoiding the clinic or social services.

Sometimes the fear and changed behavior was not in direct response to an enforcement action or the inauguration but moreso just generalized fear—or ‘paralysis’ like Brian mentioned--that affected health-care seeking. One of the Community Health Workers at CHASS works with diabetes patients and believes that many of her clients’ diabetes management has suffered in the post-election period.

*“I have a patient that barely ever leaves her house. She’s just scared, she’s really scared…[the fear] makes their [A1C] numbers go through the roof. Because they’re stressed out all the time. They’re always looking over their shoulder…sometimes they’ll go a week or two without medication because they don’t feel safe enough to come out.”*

In addition to this example from a diabetes patient, another staff member noted that many of their pregnant patients feel unable to attend prenatal appointments because they do not feel safe traveling to and from the clinic. Laura is a referral coordinator at the Detroit FQHC and recounts her frustrations trying to get pregnant women in for their prenatal appointments:

*“It’s very hard. We’ve been seeing a change in this past year with us scheduling these appointments. Patients that need these ultrasounds…They’re constantly not showing up for their ultrasounds. They’re rescheduling and everything, it’s because, you know, of the immigration status right now. They’re scared to drive, most of these ladies have an expired driver’s license for years and they don’t want to risk it. They don’t even want to risk coming to the clinic anymore because they don’t have no other transportation but theirselves and they’re scared to get out there in the street with an expired driver’s license cause they hear that the immigration is out a lot more than ever.”*

The aforementioned examples clearly illustrate the direct relationship between increased fear (generalized or targeted) and healthcare utilization, potentially resulting in worse health outcomes. In many cases, this is partly due to state-level policies that inhibit undocumented individuals from obtaining a driver’s license and thus make travelling by car more difficult. For the Community Health Worker’s client with diabetes, the decision to adopt behaviors that would protect herself from deportation and separation from her family (i.e. staying inside her house) resulted in poor health outcomes. For Laura’s clients, it resulted in avoiding prenatal care, a service that is effective in the prevention of poor maternal and child health outcomes.

Staff perceived that the fear also had an impact on utilization of public benefits. Among staff members that worked on enrolling immigrant clients in social services or public benefits, many reported that clients were less willing to enroll in government services for fear of exposing themselves to the federal government. Principally, the concern was that providing their address and contact information might allow ICE to find them and deport them. Additionally, given that the Trump administration proposed that immigrants who received public benefits would be prohibited from having a visa or obtaining citizenship—and there were rumors about this before it was officially proposed--some immigrant clients chose to avoid such services to retain the possibility of a more secure place in the U.S. through a visa or citizenship. Jimena is the WIC specialist at the Detroit FQHC and she describes these concerns:

*“We help them a lot with their income, we help them with services like Medicaid. WIC is a very important for them, well, it was…Back in the year or so they stopped coming to their appointments. We have clients who would call us and tell us they don’t want nothing to do with WIC only because of immigration.”*

Among staff that worked more intimately with immigrant families (i.e. case workers, community health workers), they heard that some of the behavior changes also affected elements of health beyond utilization of clinic or social services. For example, Adriana, a Community Health Worker in Detroit, details how this fear and anxiety related to family separation could affect food insecurity:

*“I think that there's a real fear of being stopped. Of being, you know, just the fact that racial profiling does exist, you know, feeling like you always have to look over your shoulder. Those things really do affect how people function. There are people like I mentioned, you know, they're stressed. They're depressed. They have anxiety. People that don't want to come out of their house. People that are afraid to go shopping for groceries because they don't want to put themselves at risk.”*

Mia, working as an enrollment specialist at the non-profit, echoed similar issues with the families she worked with.

*“I had another family that what they do is, like, they go food shopping once a week…they get as much as they can...they are in their home and they only go out to take the kids to school or to work…and if they run out of food in the middle of the week for whatever reason they have to wait…the kids have to wait…you’re a child and you like can’t eat anything. Food has run out and your parents are telling you ‘well we have to wait.’”*

Both of these quotes reflect that the fear in the post-election period is impacting immigrant families’ ability to get food and is potentially causing food insecurity for some families. In addition to food insecurity, we heard from staff that immigrant families are avoiding recreation at public parks and avoiding certain public social events. These decisions, made to limit exposure to the possibility of deportation, have a potential impact on mental and physical health.

Finally, the fear created new issues that staff were needing to respond to. Participants noted that clients were trying to prepare for possible deportation or detention by asking health and social service providers about arranging for power of attorney and ensuring their children would be safely cared for if they were deported. Isabel is a patient advocate at the Detroit FQHC and had lot of experience with her clients asking about power of attorney:

*“We saw in the beginning, what I would say from the inauguration till probably all last year, especially the first 6 months, all kind of letters and affidavits giving, guardianship and just people’s rights to a neighbor. Sometimes you don’t even know these people, and they’re giving kids to them. because their kids are U.S. citizens and the neighbors are U.S. citizens. [They say] ‘if they come for me I just want to make sure my kids are ok.’ They don’t even really know these people!”*

These types of power of attorney documents giving parental rights to other adults need to be renewed every 6 months. Staff members—particularly those bilingual and bicultural staff members who were seen as a community resource--shared that this is an issue that they are continually asked about. They noted that while power of attorney documents could help children in the case of a deportation, it also served as an added burden for staff and a chronic stressor for parents who were reminded that they could be removed at any moment from their children.

## Discussion

We interviewed 28 staff members at three sites across Southeastern Michigan and found that they perceived that their immigrant clients’ health and social well-being had been decisively impacted by the changes since the 2016 election. Specifically, staff noted that there was increased and pervasive fear, fractures in community cohesion, and an impact on healthcare utilization and health-related behaviors. In this section, we contextualize these findings and make recommendations for future research and public health intervention addressing these issues.

Our research findings builds upon a growing body of research that has documented the impact that recent immigration policy changes have on immigrant families. Several clinicians and news articles have documented an increase in no-shows among immigrants [2]. Additionally, some recent research shows that immigrants are unenrolling from some government assistance programs [21]. For example, one recent study of 35,000 mothers in five U.S. cities showed that Supplemental Nutrition Assistance Program (SNAP) enrollment has recently declined among immigrant families after a decade of increases [22]. Our research supports these findings and extends them by showing that changes since the election are also contributing to food insecurity and limited mobility.

These findings also connect with the growing body of literature on how immigration enforcement impacts health of immigrants. Other studies have shown that immigration raids or the implementation of a specific policy impacts health [3–7, 12, 23]. Our study, on the other hand, focuses on how the socio-political climate after the 2016 election has created conditions for immigrant families that have a negative effect on health. The stress and fear described in our data and these other study have the potential to impact health through three pathways: 1. behavioral-immigrants change health-seeking behaviors to avoid being detained, 2. psychological-the fear and stress has a major impact on adult and children’s emotional health, 3. physiological-the chronic stress prompts physiological responses that play a role in wear and tear on the body [24]. While each of these hypothesized pathways have not been fully researched, this body of work is beginning to build the evidence base for the different ways in which our immigration enforcement policies—and the stress and fear they cause—impact health outcomes for immigrant populations.

More research is needed to better understand how the 2016 election is impacting families. Follow up research should better understand the lived experience of immigrants during this period of time from their own perspective. While interviewing staff members allowed for us to have a more institutional-level view of how this is impacting immigrants, it is also important to document this time period in the voice of immigrants. We did confirm our findings with immigrant clients, but future research could take a narrative or phenomenological approach to better understand the lived experience of undocumented immigrants in this climate. Further, quantitative research is needed to see how mental and physical health outcomes—as well as psychosocial metrics of fear of deportation or social support—have changed for immigrants before and after the 2016 election. Finally, it will be important to examine quantitatively the population prevalence of the increased fear and behavior change we documented in this study as well as which health outcomes are more significantly correlated with immigration-related fear.

Our findings suggest that it is important for clinics and social service providers to develop new or expanded strategies to help immigrant families overcome some of the issues raised. For example, several staff members noted the fear that some clients have of traveling to and from a clinic. Therefore, organizations should consider expanded transportation or possibly models of telemedicine. Additionally, community health worker models could help bridge the gap between a clinic and an individual in their home by offering home visits or otherwise connecting with them off-site.

It is important for health and social service organizations—who often receive their funding from the government—to recognize how government immigration enforcement policies and actions are playing a role in their clients’ health and well-being. While leadership at these organizations may be reluctant to jeopardize funding, it is essential that they advocate on behalf of their clients by sharing stories of impact with policy-makers. Too often, immigration policy is made by those who have little interaction with immigrants. Health and social service organizations can help amplify the voices of immigrant families to help ensure that their health and well-being—and human rights—are at the forefront of policy discussions.

### Limitations

While our exploratory study provides novel findings on the impact of the 2016 election on immigrant health and social well-being, findings should be considered with several limitations in mind. First, we used a convenience sample only in two specific regions of Southeastern Michigan, these findings may not apply to other settings in Michigan or elsewhere. Second, staffs recall of events may have been biased and they may have assumed broader patterns based on the anecdote of one or two clients. While we attempted to buffer this concern by confirming findings with immigrant clients and groups of staff, we much still recognize this possible limitation. Finally, given that these interviews took place at participants place of employment, participants may have been biased to answer questions how they imagined their supervisors wanted them to. We did attempt to minimize this possibility during the informed consent procedures.

## Conclusions

Elections have wide and far reaching consequences. Based on our findings, immigrant families in Southeast Michigan are being negatively impacted by the changes caused by the 2016 election. Health and social services providers may need to adapt their services to this changing socio-political environment and advocate on behalf of immigration and health policies that facilitate health for immigrant families.

## Data Availability

The data generated and analyzed for the current study are not publicly available due the sensitive nature of the topic area and the potential for deductive disclosure due to the fact that this is qualitative data. But, the transcripts are available from the corresponding author on reasonable request.

## Abbreviations

CBP: Customs and Border Patrol
CBPR: Community-Based Participatory Research
CHASS: Community Health and Social Services Center
FQHC: Federally Qualified Health Center
ICE: Immigration and Customs Enforcement
SNAP: Supplemental Nutrition Assistance Program
WIC: Women, Infants, and Children
